# Neurotransmitter receptor densities are associated with changes in regional Cerebral blood flow during clinical ongoing pain

**DOI:** 10.1002/hbm.25999

**Published:** 2022-07-07

**Authors:** Alexandros Vamvakas, Timothy Lawn, Mattia Veronese, Steven C. R. Williams, Ioannis Tsougos, Matthew A. Howard

**Affiliations:** ^1^ Medical Physics Department, Medical School University of Thessaly Larisa Greece; ^2^ Department of Neuroimaging, Institute of Psychiatry, Psychology and Neuroscience King's College London London UK

**Keywords:** Allen brain atlas, arterial spin labelling, neurotransmitter receptors, osteoarthritis, pain, positron emission tomography

## Abstract

Arterial spin labelling (ASL) plays an increasingly important role in neuroimaging pain research but does not provide molecular insights regarding how regional cerebral blood flow (rCBF) relates to underlying neurotransmission. Here, we integrate ASL with positron emission tomography (PET) and brain transcriptome data to investigate the molecular substrates of rCBF underlying clinically relevant pain states. Two data sets, representing acute and chronic ongoing pain respectively, were utilised to quantify changes in rCBF; one examining pre‐surgical versus post‐surgical pain, and the second comparing patients with painful hand Osteoarthritis to a group of matched controls. We implemented a whole‐brain spatial correlation analysis to explore associations between change in rCBF (ΔCBF) and neurotransmitter receptor distributions derived from normative PET templates. Additionally, we utilised transcriptomic data from the Allen Brain Atlas to inform distributions of receptor expression. Both datasets presented significant correlations of ΔCBF with the μ‐opioid and dopamine‐D2 receptor expressions, which play fundamental roles in brain activity associated with pain experiences. ΔCBF also correlated with the gene expression distributions of several receptors involved in pain processing. Overall, this is the first study illustrating the molecular basis of ongoing pain ASL indices and emphasises the potential of rCBF as a biomarker in pain research.

## Graphical abstract

Arterial spin labelling (ASL) plays an increasingly important role in neuroimaging pain research but does not provide molecular insights regarding how rCBF relates to underlying neurotransmission. Here, we integrate ASL with PET and brain transcriptome data to investigate the molecular substrates of rCBF, underlying clinically relevant pain states. Both datasets presented significant correlations of ΔCBF with the μ‐opioid and dopamine‐D2 receptor expressions, which play fundamental roles in brain activity associated with pain experiences, as well as with the gene expression distributions of several receptors involved in pain processing.

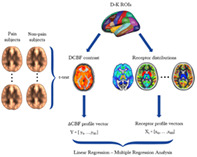

**Abbreviations**
ABA: Allen Brain Atlas
ASL: arterial spin labelling
BOLD: blood oxygen level dependent
BPnd: binding potential
CLBP: chronic low back pain
CMC: carpometacarpal
CBF: cerebral blood flow
CNS: central nervous system
DOR: δ‐opioid receptor
fMRI: functional magnetic resonance imaging
FWE: familywise error
KOR: κ‐opioid receptor
MNI: Montreal Neurological Institute
MOR: μ‐opioid receptor
mRNA: messenger ribonucleic acid
NNT: number needed to treat
NRS: numerical rating scale
NSAID: nonsteroidal anti‐inflammatory drug
PAG: periaqueductal grey
PET: Positron emission tomography
pCASL: pseudo‐continuous arterial spin labelling
TME: third molar extraction
VOI: volume of interest
OA: osteoarthritis
VAS: visual analogue scale
VDP: variance decomposition proportion
VIF: variance inflation factor
5‐HT: 5‐hydroxytryptamine

## 1 INTRODUCTION

Persistent pain is a major health problem, affecting the quality of millions of lives globally and imposing a burden on healthcare systems. Despite recent advances in our mechanistic understanding of pain, there remains a need for novel, efficacious treatments for persistent pain (Woolf, 2020). Major limitations of existing therapies include high number‐needed‐to‐treat rates (NNT) and the short‐term sustainability of treatment effects (Walsh & Stocks, 2017; Woolf, 2020). Moreover, many analgesic pharmacotherapies are associated with a variety of side effects resulting in a narrow therapeutic window. For example, while nonsteroidal anti‐inflammatory drugs (NSAIDs) offer some benefit, they increase risks of ischemic cardiovascular events and can impair renal function (Walsh & Stocks, 2017). Opiates, the prototypical analgesic, show little sustained benefit for chronic pain and are associated with serious gastrointestinal and cognitive side effects as well as risk of abuse and addiction (Walsh & Stocks, 2017).

Historically, pharmacological studies have relied entirely upon participants' self‐reports to quantify their pain experiences, but interindividual differences in pain responses have hampered patient stratification and development of novel treatments (Howard et al., 2012). Chronic pain had been viewed as ‘acute pain that is lasting too long’, with prolonged nociceptive input from sensitised nociceptors or damaged nerve fibres (Schmidt‐Wilcke, 2015). However, activation of nociceptors is neither sufficient nor necessary to produce a pain experience (Ossipov et al., 2010). For example, severity of joint damage in osteoarthritis correlates only weakly with the experienced pain (Steen Pettersen et al., 2019) and a given intensity of noxious stimulation can produce vastly diverging levels of reported pain (Coghill et al., 2003). Additionally, chronic pain patients often suffer psychiatric comorbidities including depression and anxiety (Schmidt‐Wilcke, 2015), while emotional state, context and prior experiences can profoundly alter the pain experience (Ossipov et al., 2010). Collectively, these highlight the importance of the central nervous system (CNS) in not only the conversion of nociceptive input into conscious pain perception, but also its complex modulation (Schmidt‐Wilcke, 2015). Therefore, methods to unravel complex supraspinal mechanisms that occur to amplify and maintain pain are critical to both our understanding of, and ability to treat, chronic pain conditions (Martucci & Mackey, 2016).

Whole‐brain neuroimaging techniques, such as Blood Oxygen Level Dependent (BOLD) functional Magnetic Resonance Imaging (fMRI), have provided insight into how the coordinated activity across multiple brain regions, referred to as ‘functional connectivity’, contribute to pain perception and its modulation (Necka et al., 2019). Beyond evoked pain paradigms, resting‐state BOLD has been also used to collect information about the ‘natural state’ of brain activity in order to identify differences in functional connectivity of medium‐term brain activity in chronic pain individuals (Harvey & Wise, 2012; Martucci & Mackey, 2016). Arterial Spin Labelling (ASL) is another fMRI technique gaining interest in pain research (Harvey & Wise, 2012). This approach allows for the quantification of regional cerebral blood flow (rCBF) in absolute physiological units, as a surrogate marker of neural activity. More importantly, ASL is more sensitive in capturing low‐frequency signal fluctuations compared to evoked‐response BOLD fMRI, making it well suited to characterise brain activity associated with ongoing or spontaneous pain observed in numerous pain phenotypes including postsurgical, orofacial neuropathic and musculoskeletal pain (Harvey & Wise, 2012; Howard et al., 2012; Loggia et al., 2019; Wasan et al., 2011; Youssef et al., 2014).

Despite the utility of these techniques for understanding the neural correlates of both acute and chronic pain conditions, they are inherently unable to delineate the neurochemical substrates underlying the fMRI signal (Sander & Hesse, 2017; Schmidt‐Wilcke, 2015). Analgesic drugs mediate their effects through modulating neurotransmitter systems at the molecular level. As such, there remains a gap between the significant advances in understanding pain processing indexed by neurovascular coupling and the targeting or development of existing or novel treatments to these molecular systems. Integration of Positron emission tomography (PET) offers an opportunity to probe these molecular systems using selective radiotracers, and highlight the neurochemical signatures involved in the functional networks indexed by fMRI (Khalili‐Mahani et al., 2017; Loggia et al., 2019; Sander et al., 2020). Wey et al. (2014) utilised simultaneous PET/fMRI to directly correlate neuroreceptor occupancy with regional haemodynamic changes in pressure pain and showed that pain induced changes in opioidergic neurotransmission contribute a significant component of the fMRI signal. Karjalainen et al. have collected PET/fMRI data from healthy participants to illustrate the role of opioid and dopamine systems in nociceptive processing of vicarious pain stimulation (Karjalainen et al., 2017). However, hybrid PET/fMRI provides only relatively inflexible paradigm designs, mainly constrained by the prohibitive costs, hardware demands and invasiveness (Khalili‐Mahani et al., 2017; Loggia et al., 2019). Moreover, depending on the radiotracer, a PET scan can be only selective for one neurotransmitter at the time and, due to radioactivity exposure and tracer availability, therefore only a few chemicals can be measured in a subject at a given time. To overcome limitations posed by on‐site PET acquisition, exploitation of binding potential (BPnd) templates from individual PET studies has become increasingly popular to inform neurotransmitter related activity, assuming its magnitude to be a linear function of receptor spatial distribution and availability across brain regions (Sander et al., 2020). This approach has been efficiently used in other fields of neuroimaging to map functional haemodynamic activation induced by different compounds onto distributions of their relevant target receptors (Dipasquale et al., 2019; Dipasquale et al., 2022; Dukart et al., 2018; Lawn et al., 2022; Selvaggi et al., 2019).

Despite the utility of ASL in characterising ongoing pain, to date no study has attempted to explore its underlying neurochemical basis. In line with the previous reports, here, we hypothesise that the spatial distribution of neurotransmitter receptor densities quantified by PET can inform the pain related ASL signal change to gain insight into the molecular substrates of ongoing pain. To test this, we explored associations of rCBF differences of pain versus non‐pain conditions with available normative BPnd templates of the μ‐opioid, dopamine D2 and 5‐hydroxytryptamine (5‐HT) receptor subtypes. These were selected given their important, yet diverse, roles in pain processing and endogenous pain modulation. We also incorporate receptors' messenger ribonucleic acid (mRNA) expression profiles, extracted from the Allen Brain Atlas (ABA), to inform the underlying receptor distribution by means of transcriptomics. The methodology is applied to two individual datasets of well‐established pain models, which were previously utilised in the respective studies of Howard et al. (Howard et al., 2011; Howard et al., 2012), in order to extent these reports, by exploring the neurochemical basis of the specific pain‐related rCBF changes identified: (i) acute post‐operative ongoing pain following lower jaw third molar extraction (TME; Howard et al., 2011) and (ii) chronic pain secondary to hand osteoarthritis (OA; Howard et al., 2012). To the best of our knowledge, this is the first study examining the relationship of ASL‐derived CBF changes with neurotransmitter receptors profiles and mRNA expression distributions in spontaneous and persistent, clinically relevant pain.

## 2 MATERIALS AND METHODS

### 2.1 TME participants and study design

The study was approved by King's College Hospital NHS Research ethics committee (Ref 07/H0808/115). Sixteen right‐handed, healthy, male volunteers aged 20–41 (mean age = 26.4 years) provided written informed consent to participate. All participants were examined by oral surgeons at the King's College London Dental Institute and diagnosed with bilateral recurrent pericoronitis. Participants were invited to participate in the study in response to a University circular email requesting patients requiring wisdom tooth removal or following referral from their primary care dentist to the dental institute for third molar extraction. All participants fulfilled NICE (2000) guidelines for recommended extraction of lower jaw left and right third molars (Howard et al., 2011). Female participants were excluded due to possible variability induced by the phase of the menstrual cycle on cerebral haemodynamics and postsurgical pain responses (Teepker et al., 2010). A complete review of inclusion and exclusion criteria is provided in Supplemental Part A1.

Participants were scanned on five separate occasions (S1–S5); screening/familiarisation (S1), pre‐surgical (S2) and post‐surgical sessions (S3) for the first extraction and pre‐surgical (S4) and postsurgical (S5) sessions for the second extraction. An interval of at least 2 weeks separated S3 and S4, to ensure that participants had completely recovered from their first surgery and were not experiencing any residual pain. MR examinations during sessions S2–S5 were identical, while the order of left and right tooth extraction was balanced and pseudo‐randomised across the group. Post‐surgical pain scanning sessions were commenced following recordings of three consecutive visual analogue scale (VAS) scores greater than or equal to 30/100 on a 100 mm pen and paper VAS, each spaced 10 min apart. During all scanning sessions, estimates of ongoing pain intensity were obtained using a computerised VAS, anchored with ‘no pain’ and ‘worst pain imaginable’, displayed on a screen visible to participants at the foot of the scanner bed. Computerised VAS scores had a range of 0–100, with identical anchors (Howard et al., 2011).

### 2.2 OA participants and study design

The study was approved by the local NHS research ethics committee (Ref 07/H0807/69) and 16 right‐handed postmenopausal female subjects (with a mean age of 60.8 years) who fulfilled the American College of Rheumatology criteria for carpometacarpal (CMC) OA in their dominant (right) hand and 17 age‐ and sex‐matched controls (with a mean age of 64.7 years) provided written informed consent to participate in the study (Howard et al., 2012). Importantly, OA patients were included only if the duration of their OA pain was greater than 6 months and they did not have severe pain elsewhere in the body. A complete review of the study's inclusion and exclusion criteria is provided in Supplemental Part A2.

The study consisted of two identical sessions, separated by a minimum of 7 days and a maximum of 21 days. Each session involved a screening and familiarisation stage prior to MRI. OA pain intensity estimates where acquired prior to and following each MRI session using a numerical rating scale (NRS) ranging from 0 (no pain) to 10 (worst pain imaginable; Howard et al., 2012).

### 2.3 MRI acquisition and pre‐processing

Imaging was performed on a 3 T Signa HDx whole‐body MR imaging system (General Electric, USA) fitted with an 8‐channel, phased‐array receive‐only head coil. High‐resolution T1‐ and T2‐weighted MR structural sequences were acquired for radiological assessment and image registration, using 3D spoiled gradient recalled (resolution = 1 × 1 × 1 mm) and fast spin echo sequences, respectively. Resting‐state rCBF measurements were made using pseudo‐continuous ASL (pCASL), using an irradiation time of 1.5 s and post‐labelling delay of 1.5 s. pCASL images were acquired using a single‐shot, 3D Fast Spin Echo readout resulting in whole‐brain blood flow maps, with scanning parameters as follows: time to echo 32 ms, repetition time 5500 ms; echo train length 64, matrix size 48 × 64 × 60, field of view 18 × 24 × 18 cm, and number of excitations 3 and spatial resolution of 1 × 1 × 3 mm.

Each TME participant was involved in four sessions; two pain‐free and two during post‐surgical pain. Each OA and control participant was scanned twice. Each MRI session comprised of six consecutive pCASL scans for TME and two consecutive pCASL scans for OA and control participants, respectively. The multiple scans were acquired because ASL is an inherently low signal to noise ratio (SNR) technique, thus, averaging between scans and relevant sessions is expected to improve data quality (O'Muircheartaigh et al., 2015). TME pain data were averaged across lateralised teeth, as previous investigations (Howard et al., 2011; O'Muircheartaigh et al., 2015) had demonstrated no effects of stimulus laterality on rCBF.

Pre‐processing was performed using FSL software version 4.1.5 (http://www.fmrib.ox.ac.uk/fsl) and Statistical Parametric Mapping software SPM version 12 (http://www.fil.ion.ucl.ac.uk/spm). For each subject, all collected ASL images within and across relevant pain sessions were co‐registered with each other and a mean image generated (SPM). The T2 weighted image was skull stripped using a brain extraction tool (FSL‐BET) and the resulting brain‐only image was co‐registered with the average ASL image and used as a mask to exclude extra‐cerebral signal (SPM‐CO‐REGISTER). A nonlinear transformation was calculated between the mean ASL image and a custom ASL template in the standardised, stereotaxic co‐ordinates of the Montreal Neurological Institute (MNI; SPM‐NORMALISE). The raw images were then transformed to MNI space in one interpolation step. The resulting images were smoothed with an 8 mm full width at half maximum isotropic Gaussian kernel (SPM) and masked to include grey matter voxels only. Probabilistic grey matter images in MNI space, derived from the FSL voxel‐based morphometry toolbox, were thresholded to produce a mask which included all voxels from all subjects with a 20% likelihood of being grey matter. To account for the inter‐subject variability of global blood perfusion values, all normalised, smoothed images were scaled to have a median value of 1000. This scaling was performed to increase reproducibility and to ensure that global inter‐subject differences in CBF values did not confound later analysis (Khalili‐Mahani et al., 2017).

### 2.4 ΔCBF profiles

**FIGURE 1:**
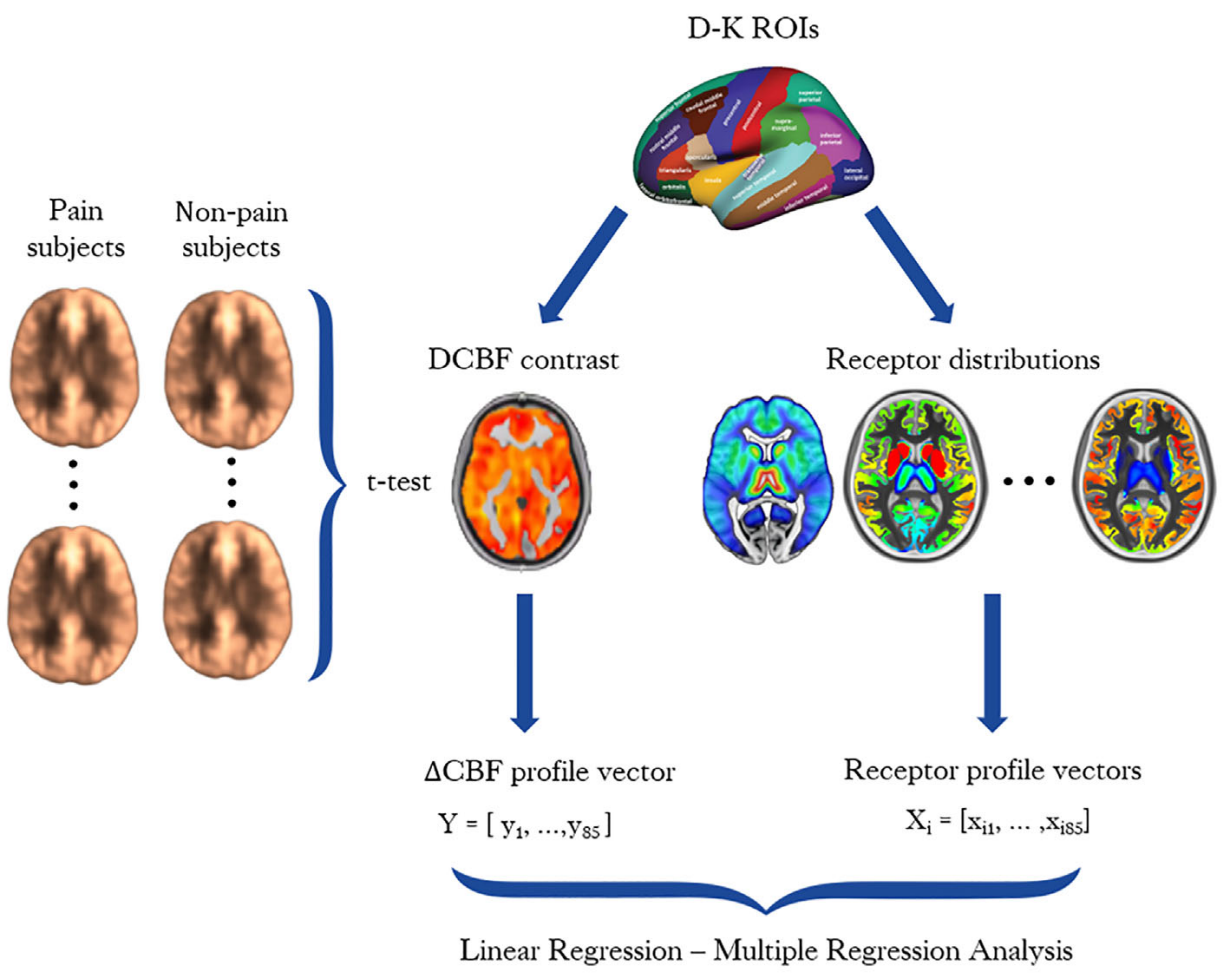
Statistical framework for ΔCBF calculation and correlation with PET templates. PET, positron emission tomography

Group level analyses to quantify CBF changes between pain versus non‐pain states was performed in SPM, using paired and independent‐group *t*‐tests for TME and OA/controls datasets, respectively. Specifically, for each dataset a voxel‐wise PAIN>NON‐PAIN t‐contrast map (ΔCBF) was calculated. A sample of axial slices of such ΔCBF maps generated is presented in Figure S1. Subsequently, the t‐contrast maps of both datasets reflecting ΔCBF were segmented into the 85 volumes of interest (VOIs) provided by the Desikan–Killiany (DK) atlas (Desikan et al., 2006). ΔCBF profile vectors for TME and OA‐controls were obtained by averaging the ΔCBF values of all voxels in each VOI (Figure 1).

Additionally, voxel‐wise PAIN > NON‐PAIN z‐contrast maps were calculated, considering cluster‐level Familywise Error rate (FWE) correction (*p* = .05, cluster defining threshold = 0.001) according to the random field theory (Worsley, 2007), to demonstrate areas of statistically significant pain‐related increases CBF. The specific maps were not utilised in the subsequent analysis, however a sample of them is presented in Figure S2, along with corresponding DK atlas VOIs, as this might be valuable to the reader. Please also refer to the previous studies of Howard et al. (2011, 2012) where a more extended analysis and discussion of the ASL findings in the specific cohorts is provided.

As it was mentioned before, in the current study, we have considered uncorrected whole‐brain t‐contrast maps and all 85 cortical and subcortical VOIs as defined in the DK atlas to test the association of ΔCBF and receptor density profiles. The rationale for including the whole brain in the analysis, rather than focus only on the regions previously known to be implicated in pain or those demonstrating a significant increase in CBF, is based on the fundamental assumption of the current study; namely, that if a neurotransmitter's activity is related to the pain stimulus, then the observed distribution of CBF change should spatially match the concentration of the respective neurotransmitter receptor within the whole brain. Thus, it is necessary to utilise all the available parcellations into the model in order to account the CBF variability across the whole‐brain, to perform an unbiased correlation for testing this hypothesis and confirming the validity of the technique. We anticipated that a whole‐brain analysis might lead to weaker associations of ΔCBF with the neurotransmitter receptors distributions, due to the inclusion of receptor‐rich regions that are not conventionally associated with pain processing. Nevertheless, beyond avoiding the statistical bias in the correlation models, there is also evidence that ongoing pain may be associated with increased blood flow in brain regions both within and outside those commonly associated with experiencing pain, for example, the somatosensory, prefrontal and insular cortices, but also the superior parietal lobule, which is part of the dorsal attention network (Schmidt‐Wilcke, 2015). Additionally, from a clinical perspective, the inclusion of all VOIs is important for comparing results between the different pain pathologies in which there may be subtly different patterns of rCBF alterations that relate to the phenotypic and demographic aspects of the cohorts in question.

### 2.5 Receptor BPnd profiles

Receptor BPnd profiles were obtained from previously published PET templates quantified from healthy subjects. Density of μ‐opioid receptor expression as revealed by [11C]carfentanil PET scans from 89 healthy volunteers (Tuominen et al., 2014) was acquired from the Neurovault collection (https://identifiers.org/neurovault.image:115126). Dopamine receptor D2/D3 expression was extracted from an independent [18F] Fallypride PET template obtained by averaging six BPnd maps of healthy young volunteers (Dunn et al., 2009). The publicly available high‐resolution in vivo atlas of four serotonin receptors, that is, 5‐HT1A, 5‐HT1B, 5‐HT2A and 5‐HT4 (https://xtra.nru.dk/FS5ht-atlas/), created from molecular high‐resolution PET scans acquired in 210 healthy individuals with different selective PET‐radioligands was also utilised (Beliveau et al., 2017). Similar to the process followed for ΔCBF profile vectors, all the available PET templates were segmented into 85 VOIs with the Desikan–Killiany atlas and region‐wise average BPnd values were calculated (Figure 1). A sample of axial slices of the PET maps utilised in the study is presented in the Figure S1.

### 2.6 mRNA expression profiles

Brain transcriptome profiles were obtained from the ΑΒΑ (https://human.brain-map.org/), an open access, multimodal atlas integrating anatomic and genomic information of the human brain (Hawrylycz et al., 2012) that can serve as a reference standard to explore relationships between gene expressions and in vivo functional imaging data (Molet & Pohl, 2013). ABA comprises microarray‐based mRNA expression values sampled over post‐mortem brain tissue from five males and one female donors between 18 and 68 years of age, with no known neuropsychiatric or neuropathological history. The anatomical sites of tissue sample acquisition are projected on a high‐resolution brain template in MNI coordinates that facilitates integration with the imaging data.

Current analysis has focused on the mRNA expressions of 34 neurotransmitter receptor subtypes for investigating the potential relationship with ΔCBF changes in a data‐driven approach, that is, μ‐, κ‐, δ‐opioids; dopamine D1–D5; adrenaline α1‐A, ‐B, ‐D; adrenaline α2‐A, ‐B, ‐C; adrenaline β1, β2, β3; serotonin 5‐HT‐1A, ‐1B, ‐1D, ‐1E, ‐1F, −2A, ‐2B, ‐2C, −3A, ‐3B, ‐3C, ‐3D, ‐3E, ‐4, ‐5A, ‐6, ‐7. For this task, the MATLAB toolbox: Multimodal Environment for Neuroimaging and Genomic Analysis (MENGA; http://www.nitrc.org/projects/menga/), which allows integration of ABA and imaging data, was utilised (Rizzo et al., 2016).

Since the between‐donors gene expression values are highly heterogeneous and tissue sampling generates variability, with the potential to compromise spatial correlations, we have focused our analysis on the left hemisphere only, for which there were more specimens available in ABA. In addition, the imaging and genomic data may present substantial spatial heterogeneity within anatomical regions of interest. While for MRI and PET data a continuous set of voxels is available, that enables averaging among a sufficient number of voxels values to obtain a representative mean per region, in case of ABA the tissue samples are provided in discrete locations within brain tissue. Similarly, the MENGA software utilised here for the imaging‐transcriptomic correlation analysis, performs a discrete resampling of CBF to match the exact anatomical locations of tissue sampling. Accordingly, efficient quantification of CBF and mRNA expressions is required to perform the correlation analysis; larger VOIs are needed, particularly for cortical regions, to obtain sufficient data per region to compensate for this inherent spatial heterogeneity. Thus, both ΔCBF contrast maps and ABA mRNA samples have been grouped over 15 coarse ABA regions. These include a combination of cortical and subcortical VOIs, comprised of whole volume delineations of frontal, temporal, parietal and occipital lobes, as well as the brainstem, amygdala, thalamus, hippocampus, cerebellum, cingulate gyrus, insula, striatum, globus pallidus, claustrum and basal forebrain.

### 2.7 Statistical analysis

Paired‐samples *t*‐tests and one‐sample *t*‐tests were used to assess statistically significant differences of the pain intensity ratings across pain and pain versus pain‐free sessions, respectively, for both TME and OA‐Controls datasets.

Linear regression models (Pearson correlation) were built in MATLAB 2019b (https://www.mathworks.com/), to correlate ΔCBF responses of TME Pain versus NoPain and OA versus Pain‐free control datasets with regional receptor BPnd distributions (explanatory variables). Initially, the normality of regression residuals' distribution was assessed with Shapiro–Wilk test for normality. In cases of highly skewed distributions, a log‐linear transformation was applied. A cut‐off value of 10 times the mean Cook's distance was used to exclude extreme observations. Non‐parametric Spearman's correlations between ΔCBF profiles and receptor BPnd profiles were also performed. Additionally, a stepwise regression function was used to build multiple linear regression models, while Variance Inflation Factor (VIF), and Variance Decomposition Proportion (VDP), were used as additional diagnostic tools to correct for multicollinearity between BPnd variables, considering thresholds of VIF < 3 and VDP < 0.9 for Condition Index > 30 (Hair et al., 2010). Bonferroni correction was considered to assess statistical significance (Figure 1).

The univariate analysis utility in the MENGA toolbox was used for correlating ΔCBF with receptor gene expressions. The univariate cross‐correlation analysis consists of the weighted regression of CBF contrast images and mRNA data for each donor. The weights are defined as the ratio of the number of samples in each region over the variability of the image data in that region for each subject. Specifically, the higher the number of samples, the smaller is the expected variability in an VOI. The linear regression results are expressed in terms of squared Pearson‧s correlation coefficients (*R* ^2^), directionality of the correlation (+1 or −1), and the number of times (out of 6 matches) for which MENGA finds a positive or negative correlation (Rizzo et al., 2016). The between‐donors autocorrelation for both imaging and genomic data is also returned as a metric to assess the consistency of mRNA expressions among donors.

## 3 RESULTS

### 3.1 Pain intensity ratings analysis

**FIGURE 2:**
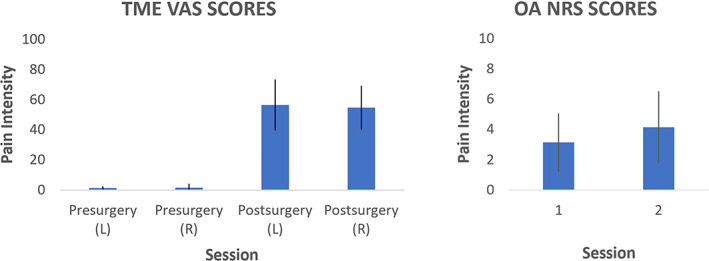
Bar plots showing the mean ± SD pain intensity ratings of all subjects pCASL scans within each session, as indexed by VAS for TME participants (left chart) and NRS for OA patients (right chart). NRS, numerical rating scale; OA, osteoarthritis; TME, third molar extraction; VAS, visual analogue scale

TME Participants reported significant increases in VAS‐derived subjectively reported pain following TME, compared to pain‐free pre‐surgical sessions (0–100 VAS scores averaged across all pCASL scans: Pre‐surgery mean ± SD = 1.67 ± 2.02, Post‐surgery = 55.56 ± 15.77, *p* < .001). Pain intensity VAS scores following extraction of left, compared to right, third molars did not differ (*p* = .97; Figure 2). OA NRS estimates of ongoing pain (mean ± SD = 3.65 ± 2.21) differed significantly compared from controls, that presented zero mean NRS measurement in all scans (*p* < .001). We observed NRS scores in the OA group to be significantly higher in session 2 (mean ± SD = 4.15 ± 2.37) than in session 1 (mean ± SD = 3.15 ± 1.92, *p* < .001; Figure 2).

### 3.2 rCBF group level analysis

*TME*: Group level analysis revealed a distributed network of brain regions with significant increases in rCBF following the extraction of left and right third molars, compared to pain‐free pre‐surgical scans in the same participants. Regions showing rCBF increases included, but were not limited to thalamus, primary and secondary somatosensory cortices, anterior and posterior insula, anterior cingulate cortex and mibrain. Post‐surgical decreases in CBF were not observed, and there were no significant differences of rCBF between cerebral hemispheres, in either pre‐surgical or post‐surgical scanning sessions following either left or right TME.

*OA*: A distributed network of brain regions demonstrated local increases in CBF in participants with OA compared to matched controls, largely lateralised to the left hemisphere contralateral to the painful joint. There were no increases in rCBF identified in the control group compared to the OA group.

A sample of cluster‐corrected *z*‐score maps showing the abovementioned significant increases in regional CBF along with corresponding DK VOIs are provided in the Figure S2. Additionally, the ΔCBF profiles, in terms of average *t*‐scores of all DK VOIs, that were utilised for the spatial correlation with receptor BPnd profiles, are presented Table S1.

### 3.3 Linear correlations

**FIGURE 3:**
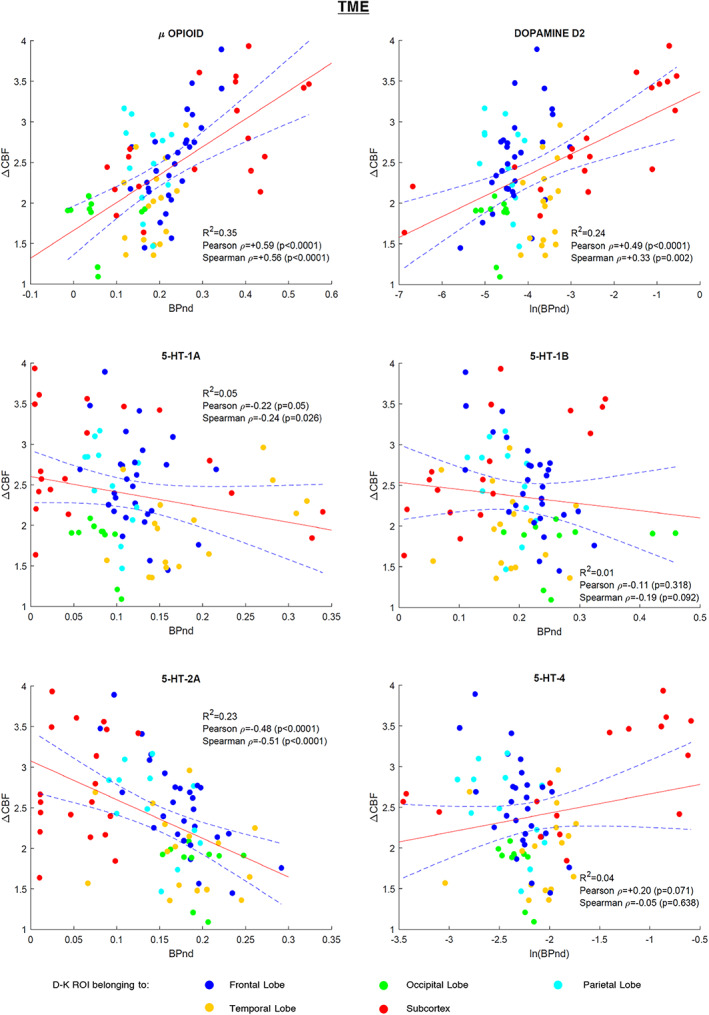
TME study regression plots: Scatterplots of the 85 VOIs (dots) displaying the relationship of the average regional CBF change (ΔCBF) between pain and non‐pain states of TME dataset with average regional BPnd values of the six receptor templates utilised. The linear regression curve (red line) and 95% confidence bounds (dashed lines) are shown. The dots are presented color‐coded as shown in the figure legend to provide a coarse positioning of the VOIs into the brain. BPnd, binding potential; CBF, cerebral blood flow; TME, third molar extraction; VOI, volumes of interest

ΔCBF statistically significant correlations (*t*‐test, *p* < .05) were found with μ‐opioid, D2, and 5‐HT‐2A receptor distributions in the TME dataset. μ‐Opioid demonstrated the strongest positive association [*R* ^2^ = 0.35, Pearson rho = +0.59, Spearman rho = +0.56] followed by D2 [*R* ^2^ = 0.24, Pearson rho = +0.49, Spearman rho = +0.33], while a strong negative association was observed for 5‐HT‐2A [*R* ^2^ = 0.23, Pearson rho = −0.48, Spearman rho = −0.51]. Weak negative associations were observed for 5‐HT‐1A [*R* ^2^ = 0.05, Pearson rho = −0.22, Spearman rho = −0.24] and 5‐HT‐1B [*R* ^2^ = 0.01, Pearson rho = −0,11, Spearman rho = −0.19] and a weak positive correlation for 5‐HT‐4 [*R* ^2^ = 0.04, Pearson rho = +0.20, Spearman rho = −0.05]. The results are summarised in Figure 3.

**FIGURE 4:**
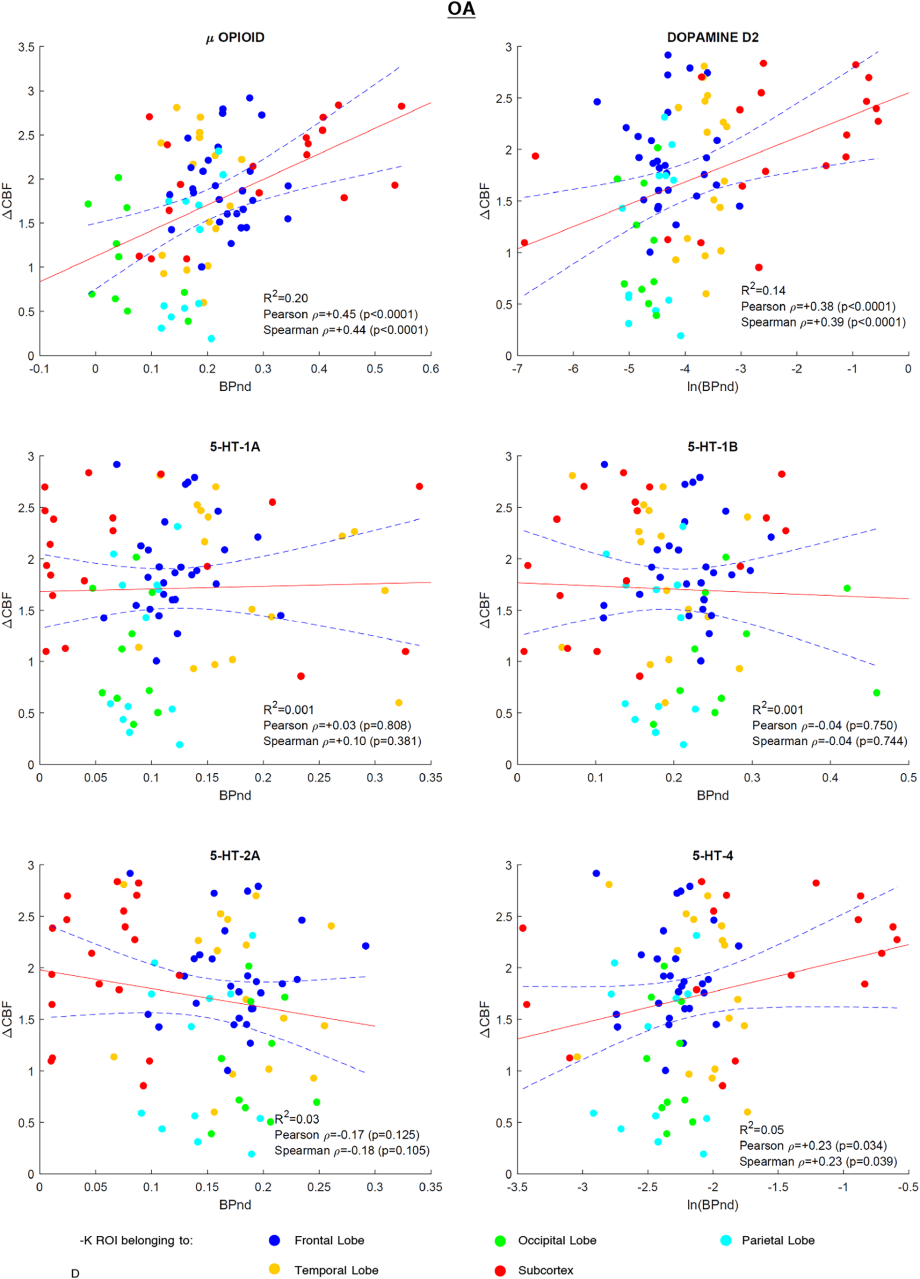
OA regression plots: Scatterplots of the 85 VOIs (dots) displaying the relationship of the average regional CBF change (ΔCBF) between pain and nonpain states of OA and controls datasets with average regional BPnd values of the six receptor templates utilised. The linear regression curve (red line) and 95% confidence bounds (dashed lines) are shown. The dots are presented color‐coded as shown in the figure legend to provide a coarse positioning of the VOIs into the brain. BPnd, binding potential; CBF, cerebral blood flow; OA, osteoarthritis; VOI, volumes of interest

Statistically significant associations (*p* < .05) were observed between OA versus control rCBF differences and μ‐opioid [*R* ^2^ = 0.20, Pearson rho = +0.45, Spearman rho = +0.44], D2 [*R* ^2^ = 0.14, Pearson rho = +0.38, Spearman rho = +0.39]. *R* ^2^ and rho values were consistently lower in the OA versus control dataset, compared to the TME data analysis. In contrast with TME data, no statistically significant associations with the serotonin maps were found. The results are shown in Figure 4.

### 3.4 Multiple linear regression

Multiple linear regression models for TME and OA datasets included all receptor templates except for 5‐HT‐4 that was found redundant. The adjusted *R*‐squared values were *R* ^2^ = 0.44 and *R* ^2^ = 0.16, for TME and OA, respectively. Statistical significance of the regressors was used to evaluate the contributions of each receptor to the multivariate model fits. In TME model the μ‐opioid, D2 and 5‐HT‐2A survived Bonferroni correction (*p* = .05/5 = .01), exactly replicating results of univariate analysis. In the OA‐control model, only the μ‐opioid receptor reached significance.

### 3.5 mRNA correlation

Within the genomic‐imaging correlation analysis performed in MENGA, only 18 out of 35 genes examined presented a sufficiently high between‐donor mRNA expression consistency (auto‐correlation values *R* ^2^ > 0.40) to be reported (detailed in Table 1). The directionality of the correlations was found to be consistent in both TME and OA datasets, respectively, however their cross‐correlation values with rCBF presented great variability between the two datasets. Statistically significant correlations (*p* < .05) were observed for OPR‐K1 in TME, DR‐D2 in OA, ADR‐A‐2A in OA and 5‐HTR‐5A in TME and OA.

**TABLE 1:** mRNA correlation results

Gene group	Gene	Gene auto‐correlation	TME cross‐correlation		OA cross‐correlation		Direction of correlation
		(*R* ^2^)	(*R* ^2^)	(*p*)	(*R* ^2^)	(*p*)	
Opioid	OPR‐M1	0.82	0.11	.230	0.10	.245	+1
	OPR‐K1	0.72	0.30	.034^a^	0.23	.070	+1
Dopamine	DR‐D1	0.69	0.16	.140	0.15	.154	+1
	DR‐D2	0.88	0.12	.207	0.36	.018^a^	+1
	DR‐D3	0.44	0.19	.104	0.04	.475	+1
	DR‐D5	0.44	0.06	.379	0.10	.245	−1
Adrenaline	ADR‐A‐1A	0.45	0.16	.140	0.05	.431	+1
	ADR‐A‐1B	0.94	0.02	.619	0.19	.104	−1
	ADR‐A‐2A	0.78	0.13	.188	0.31	.029^a^	+1
	ADR‐A‐2C	0.89	0.08	.307	0.06	.379	+1
	ADR‐B‐2	0.54	0.17	.129	0.21	.086	+1
Serotonin	5‐HTR‐1A	0.90	0.05	.431	0.19	.104	−1
	5‐HTR‐1E	0.82	0.06	.379	0.08	.307	−1
	5‐HTR‐2C	0.90	0.23	.070	0.09	.277	+1
	5‐HTR‐3B	0.48	0.19	.104	0.19	.104	−1
	5‐HTR‐5A	0.60	0.27	.047^a^	0.30	.034^a^	−1
	5‐HT‐4	0.73	0.07	.340	0.09	.277	+1
	5‐HTR‐7	0.80	0.26	.058	0.13	.188	+1

Abbreviations: OA, osteoarthritis; TME, third molar extraction.

^**a**^ Statistically significant correlations.

## 4 DISCUSSION

The present study demonstrates relationships between ASL‐derived rCBF indices of ongoing pain and specific receptor spatial distribution profiles obtained from PET and mRNA data within neurotransmitter systems relevant to clinical pain. Moderate to strongly significant correlations were observed between ΔCBF and both mu‐opioid and D2, while weak positive and negative correlations for the 5‐HT receptors' BPnd distributions were identified in both spontaneous (TME) and persistent (OA) pain models. Only the 5‐HT‐2A receptor showed a significant negative correlation with ΔCBF in TME. We observed stronger μ‐opioid and D2 receptor correlations with ΔCBF in the TME, compared to the OA dataset. Multiple linear regression models were found to largely replicate the univariate analysis results, but additional weaker effects of the receptors on ΔCBF were not observed in these models. The significant relationships found between receptor mRNA expressions and ΔCBF were relevant to the receptors' expected functions in pain processing, but correlation coefficient values varied between datasets and corresponding results of ΔCBF and PET templates analysis. This study provides important new evidence regarding the link between pain‐related, ASL‐derived rCBF signals with the opioidergic and dopaminergic systems, two crucial components of pain processing (DaSilva et al., 2019). Beyond acting as “proof of concept”, these findings help inform future investigation of the molecular mechanisms underlying painful experiences, namely neurotransmitter systems, which may show functionally relevant disruption in acute and chronic pain conditions, as well as modulation by treatment.

Several major differences were immediately apparent between the TME‐pain and OA datasets. Mechanistic insights from each study have been described previously (see Howard et al., 2011, 2012) but are briefly summarised here. Perhaps most notably, in the TME data, a remarkably symmetric distribution of increases in rCBF following removal of both left and right teeth was observed. By contrast, rCBF increases in OA participants were markedly lateralised, predominantly located in the left hemisphere contralateral to the painful thumb. Laterality issues aside, there were many similarities between the two datasets, namely, a distributed network of increases in rCBF only in brain regions commonly associated with the pain experience, including primary and secondary somatosensory, anterior and poster insula and anterior cingulate cortices, thalamus, and midbrain (including the Periaqueductal Grey [PAG]). rCBF increases in several of these regions have also been reported in other chronic pain cohorts, for example Chronic Low Back Pain (CLBP; Wasan et al., 2011). Quantitative comparisons between datasets were not performed here, given so many phenotypic differences between TME and OA datasets respectively, including pain phenotype (acute post‐surgical vs. persistent pain); body site (bilateral dentition vs. unilateral hand); age (young vs. older adults); sex (male vs. female); experimental design (within vs. between subject). Often it is considered more straightforward to study experimentally induced pain in healthy volunteer participants, compared to patients with chronic pain. The latter are a comparatively more heterogeneous cohort, with defining characteristics not only in terms of behaviour (inescapable, ongoing daily pain, psychological sequelae including anxiety and depression; medication use, etc.), but also brain structure (May, 2008) and brain function (Youssef et al., 2014). In view of producing transiently inescapable ongoing post‐surgical pain, we and others have argued in favour of the TME model being an excellent ‘half‐way house’ between acute and chronic pain states (Kupers & Kehlet, 2006). The reader is referred to the recent review of (Loggia et al., 2019) which provides a thorough summary of reports of rCBF changes relating to acute and chronic pain states. However, what is important in this proof‐of‐concept study is the demonstration of a relationship between the spatial distribution of neurotransmitter receptor densities, and changes in rCBF associated with individuals' pain experiences, both experimentally induced and chronic.

We acknowledge that the techniques employed here have some limitations. Spatial autocorrelation—that is, statistical dependence between neighbouring voxels/regions—is inherent within brain maps that might increase the type I error of common statistical inference frameworks. While some spatial permutation methods exist for generating spatially null models for brain maps (for a complete review see Markello & Misic, 2021), none of them are suitable for applying spatial shuffling across both hemispheres and both cortical and subcortical areas in the same model. This intrinsic limitation is due to the requirement for different distance calculation methods between corresponding parcels, thus constraining the utility of current approaches for whole brain correlation analyses. Our consideration of transcriptomic data provides additional challenges to any approach to this problem. We consider that the development of appropriate methodologies to mitigate spatial autocorrelation issues as an important future challenge for the field.

Our method exploits the availability of normative receptor templates, but it should be acknowledged that these data do not account for potential pathologic or neuroplastic changes that may be induced by chronic pain conditions, or possible effects of long‐term medication use on receptor expression profiles. In an ideal world, to maximise interpretability and validity of results, studies would make use of subject‐specific PET scans to provide precise quantification of receptor binding and directly addressing the underlying neurochemical conditions. However, broad consideration of multiple neurotransmitter systems would be financially punitive, not least largely impossible to implement given both the cost and safety limitations on repeated administration of radiotracers. The benefit of the current technique is that it can leverage existing datasets and provide insights when more expensive and complex techniques have yet to, or cannot, be employed. As such, the current approach offers significant utility as a low‐risk hypothesis generating tool at a relatively low cost.

The most important finding of our study was the significant correlation of ΔCBF with the μ‐opioid receptor profile. Arguably, this validates the techniques employed here; the μ‐opioid receptor plays a well‐established pivotal role in pain processing (Zubieta et al., 2001). Increased neural activity in opioid‐rich descending pain modulatory structures such as the rostral anterior cingulate cortex, amygdala, and PAG has been reported during placebo interventions (Skyt et al., 2020). In a previous study, placebo responders that received the opiate antagonist naloxone under blinded conditions indicated pain levels similar to those of the non‐responders, indicating that the mechanism of placebo analgesia engaged required engagement of endogenous opioid‐mediated systems (Ossipov et al., 2010). Moreover, opioid analgesics including morphine, methadone, fentanyl, and oxycodone are a cornerstone in the pharmacotherapy for pain and act primarily upon the μ‐opioid receptors (MOR; Lueptow et al., 2018). The strong association between ΔCBF and μ‐opioid receptor particularly observed in the TME dataset is in accordance with previous preclinical (Shih et al., 2012) and clinical (Karjalainen et al., 2017; Wey et al., 2014). PET/fMRI studies of acute pain conducted with the μ‐opioid selective radiotracer [11C]carfentanil, further adding credence to the ability of the techniques employed here to capture meaningful molecular relationships. Given its sensitivity to detect low‐frequency signal fluctuations, ASL has the potential characteristics to be developed as a biomarker to probe opioidergic systems, both in patient/control designs as well as ‘within‐subject’ experimental conditions, for example, drug/placebo comparisons.

Associations between ΔCBF and μ‐opioid in the OA group were weaker than those identified in the TME cohort. Although this may partially be attributed to the increased heterogeneity between OA and healthy control groups utilised in the specific model design, however the contradictory reports regarding the role of mu‐opioid receptor in mediating analgesia within chronic pain conditions remains highly equivocal (Gwilym et al., 2009). For example, two previous studies found that administration of naloxone did not block placebo effects in patients with chronic pain, suggesting that the endogenous opioid system functions differently under conditions of chronic pain (Skyt et al., 2020). Similarly, recent reports suggest that mechanisms in addition to the opioidergic system are also important in mediating placebo responses more generally (Crawford et al., 2021). Chronic pain is associated with structural and functional changes in the central nervous system that affect multiple brain structures involved in pain perception and modulation. Recent MRI and PET studies have provided insights into the maladaptive neuroplasticity related to chronic pain, such as the reduced μ‐opioid receptor availability in chronic pain disorders including rheumatoid arthritis and neuropathic pain (DaSilva et al., 2019). Furthermore, a previous study has shown increased levels of endogenous opioids in the cerebrospinal fluid of fibromyalgia patients, an indirect index of reduced μ‐opioid receptor availability, which may hold some explanatory value for the poor efficacy of opioids in fibromyalgia (Harte et al., 2018). Potentially, altered opioid neurotransmission in our OA patients might have affected the association of μ‐opioid receptor density with ΔCBF, illustrating a limitation when using normative receptor templates, as previously described.

We also sought to investigate potential monoaminergic associations with rCBF. Monoamines regulate the endogenous pain system (Bannister & Dickenson, 2016), and both peripheral and central monoaminergic dysfunction has been reported in various pain aetiologies (Bravo et al., 2019). Currently monoaminergic pharmacotherapies do not constitute a first line choice for chronic pain (e.g., in osteoarthritis; Kloppenburg et al., 2019). However, recently drugs like duloxetine have been repurposed from other neuropsychiatric conditions and are used for neuropathic pain and fibromyalgia. Such drugs are thought to impart analgesic efficacy through inhibiting the reuptake of monoamines in brain and spinal levels (Bravo et al., 2019).

Dopamine's central circuitry has an important role in pain processing, while among the five subtypes of dopamine receptors (D1–D5) in the CNS, the D1 and D2 are those most strongly implicated in pain modulation within animal models (Bravo et al., 2019). Activation of D2/D3 receptors at the spinal level induces an anti‐nociceptive effect, whereas stimulation of D1/D5 receptors is pro‐nociceptive (Bravo et al., 2019). Early evidence from human PET imaging studies have shown a strong correlation of striatal D2 receptor availability with individual variations in subjective ratings of sensory and affective qualities of persistent pain (Scott et al., 2006) in fibromyalgia (Ledermann et al., 2016). In our study, a statistically significant correlation was observed between ΔCBF and the D2 receptor in both datasets, while the D2 association follows a similar trend with μ‐opioid. Although this finding is important, however a straightforward interpretation is difficult; strong co‐localisation of μ‐opioid and D2 receptor distributions exists, and this kind of analysis has an intrinsic limitation in differentiating between co‐founding effects. In other words, we cannot be sure of the extent to which the result reflects a direct D2 receptor involvement, or a mixed effect of interactions between the opioid and dopamine systems.

We suggest that future studies should take into account these considerations and previous evidence, and particularly utilise PET/fMRI to shed further light on the opioid–dopamine system, which has been shown to play an important role in endogenous analgesia and placebo effects (Shih et al., 2012). Specifically, as most striatal neurons express both opioid and dopamine D2/D3 receptors, it is thought that dopamine produces analgesic effects via interactions with endogenous opioids in midbrain areas (Bannister & Dickenson, 2016). A recent study exploiting 7 T fMRI has shown evidence that placebo is likely mediated by the lateral PAG, an area that produces a non‐opiate mediated analgesia upon stimulation (Crawford et al., 2021). Generally, PAG that is an essential element of the descending pain modulatory system (DaSilva et al., 2019), has a dense concentration of mu‐opioid receptors but also contains a subpopulation of dopaminergic neurons that, if ablated or antagonised, attenuates the antinociceptive effects of systemic morphine. On the other hand, dopamine receptor agonists and dopamine transport inhibitors enhance the antinociceptive effects of opioids (Bannister et al., 2009). Other analgesics (e.g., gabapentinoids) that were used in combination with those having a direct effect on monoaminergic system have been reported to alleviate certain types of chronic pain (Bravo et al., 2019; Woolf, 2020). Therefore, combined therapies that modulate multiple neurotransmitter systems may offer stronger therapeutic benefit than that of systemic analgesics.

Serotonin (5‐hydroxytryptamine, 5‐HT) is a monoamine widely distributed both at the periphery and in the central nervous system. Although the peripheral pronociceptive role of 5‐HT is well established, its modulatory role at the spinal and supraspinal levels seems highly variable, depending on the type of receptor, the neural structure and pathophysiological condition, emphasising the complexity of its implications for the neurobiological mechanisms underlying nociception (Viguier et al., 2013).

In general, there are very few in vivo human PET studies quantifying 5‐HT receptor subtypes due to the lack of selective radioligands or sparsity of receptors in the brain (Martikainen et al., 2018). Among the few existing 5‐HT receptor PET templates that were used in this study, only the 5‐HT‐2A showed a moderate, but inverse association with the ΔCBF profile, particularly in the TME dataset. In a previous PET study, the 5‐HT‐2A receptor availability in the brain, and specifically in regions involved in cognitive and affective functions, was found to co‐vary strongly with the responses to long‐lasting (tonic) heat pain stimulus (Kupers et al., 2009). The authors suggested that 5‐HT‐2A has a role in pain processing but is more related with the cognitive and emotional assessment of painful stimuli rather than its implication in antinociception. We speculate that these pain excitatory effects of 5‐HT‐2A are illustrated here by the negative nature of the correlation in the TME post‐surgical pain dataset.

A previous PET study that correlated responses to the cold pressor test with 5‐HT‐1A receptor binding in the brain found 5‐HT‐1A receptors to be involved in regulation of pain‐related responses (Martikainen et al., 2007). However, the association between pain intensity and 5‐HT‐1A BPnd was also present in brain regions not conventionally associated with pain processing, possibly indicating an indirect serotoninergic effect on behavioural responses rather than a specific pain modulatory action. These findings were further validated by the same authors in a newer study (Martikainen et al., 2009) and could explain the insignificant relationship of ΔCBF and 5‐HT‐1A distributions observed in our whole‐brain analysis.

A significant correlation of 5‐HT‐1B and 5‐HT‐4 with haemodynamic responses was not observed here and there are no reports from human imaging studies addressing the role of the specific 5‐HT receptors in pain that could help us elucidate these findings. In general, the 5‐HT‐1B/D receptors are thought to have similar action in pain processing with the 5‐HT‐1A receptors, because of their homologous structure (Tao et al., 2019). Preclinical studies comparing agonists and antagonists for 5‐HT receptor subtypes, found a significant antinociceptive role for supraspinal 5‐HT‐4 and 5‐HT‐7 receptors in visceral and neuropathic pain models (Tao et al., 2019). This highlights the potential importance of these receptors in pain inhibition, which requires further investigation.

We also exploited gene expression profiles from the ABA microarray data to obtain information about receptors' spatial distributions by means of mRNA availability. In general, the directionality of the relationships between receptor expression and ΔCBF, are consistent with our ASL/PET analyses, however direct comparisons of the magnitudes of the associations between methods would be arbitrary due to their differing implementations. Of note, besides the small sample size underpinning ABA and interindividual differences between donors, mRNA expressions only approximate cellular protein levels due to post‐transcriptional regulatory mechanisms (Rizzo et al., 2016). That said, the directionality of the ΔCBF associations with both PET and mRNA templates accords with the expected functions of supraspinal receptors in pain processing. This is perhaps best illustrated here by the positive correlation of the receptors that exert inhibitory effects (e.g., μ‐opioid, D2/D3, ADR‐A‐2A/C and 5‐HT‐7) and the negative/weak correlation for receptors that are thought to facilitate pain signals or are more associated with behavioural and emotional aspects of the pain experience (e.g., D5, 5‐HTR‐1A/B, 5‐HTR‐2A and 5‐HTR‐3B; Sander & Hesse, 2017). More importantly, considerations regarding directionality relate to existing literature that has directly investigated receptor‐specific PET and haemodynamic measures at a mechanistic level, further adding credence to the ability of the techniques employed here to capture meaningful molecular relationships (Sander et al., 2020). However, it should be noted that an inverse relationship between the signs of the observed inhibitory and excitatory functional responses should be expected when comparing the two methodologies. This is because the previous studies that have made use of simultaneous or sequential PET‐MRI address the functional responses through the dynamic measurement of BPnd decrease, that is, loss of receptors' availability, as an indicator of neuronal activation. In contrast, the method implemented here assumes the magnitude of neurotransmitter activity to be proportional with receptor's spatial distribution and availability across brain regions, as indexed by the static PET templates or the transcriptomic data utilised.

Further statistically significant correlations with ΔCBF were observed for mRNA indices of kappa‐opioid and adra‐A‐2A receptors, implying a potential supplementary role in pain modulation. Though the MOR is the main target for opioid analgesics, the δ‐ (DOR) and κ‐ (KOR) opioid receptors have recently gained attention as potential targets for pain and analgesia regulation (Valentino & Volkow, 2018). The relative affinities of opioid analgesics for these receptors confer unique properties relating to mood and stress reactivity (Valentino & Volkow, 2018). Specifically, while MOR agonists produce euphoria and promote stress coping, KOR agonists produce dysphoria, stress‐like responses and negative affect, whilst agonists at DOR reduce anxiety and promote positive affect. Our findings indicate functional significance of KORs in ongoing pain and adds credence to attempts to understand how best to pharmacologically modulate the multiplicity of opioid receptors to maximise clinical utility whilst minimising adverse effects (Valentino & Volkow, 2018). Recent evidence has also demonstrated adrenergic analgesic effects imposed by pharmacologic stimulation of cortical a2A adrenoceptors in animals (Wang et al., 2017). Overall, we consider this initial evidence important as several gene candidates could play an important role for understanding neuron structural and functional alterations apparent in persistent pain conditions and serve for the development of individualised pain therapies (Denk & McMahon, 2017; Wu & Raja, 2011).

## 5 CONCLUSIONS

In conclusion, we provide a novel demonstration of the relationship between ongoing pain, as informed from ASL‐based rCBF, and a priori understanding of molecular receptor density profiles. Strong relationships were found for the μ‐opioid receptor and dopamine D2 receptor, further supporting the primacy of these neurotransmitter systems in pain processing. The methodology deployed here is a useful tool to help bridge the translational gap between the advancing knowledge gained from MRI and the neurotransmitter systems that underlie these findings. Specifically, understanding the neurotransmitter systems engaged during pain, perturbed in chronic pain, and modulated under analgesic intervention are crucial steps to producing evidence‐based precision medicine and development of novel analgesic pharmacotherapeutics.

## CONFLICT OF INTEREST

The authors declare that there is no conflict of interest.

## FUNDING STATEMENT

The authors disclosed receipt of the following financial support for the research, authorship, and/or publication of this article: Alexandros Vamvakas was supported by the Hellenic Foundation for Research and Innovation (HFRI) under the HFRI PhD Fellowship grant [fellowship number: 35]; Timothy Lawn is in receipt of a PhD studentship funded by the National Institute for Health Research (NIHR) Biomedical Research Centre at South London and Maudsley National Health Service (NHS) Foundation Trust and King's College London; Mattia Veronese is supported by MIUR, Italian Ministry for Education, under the initiatives “Departments of Excellence” (Law 232/2016), by Wellcome Trust Digital Award (no. 215747/Z/19/Z) and by the NIHR Biomedical Research Centre at South London and Maudsley NHS Foundation Trust and King's College London. Matthew A. Howard and Steven C.R. Williams are funded by a Medical Research Council Experimental Medicine Challenge Grant [grant number: MR/N026969/1] and are also supported by the NIHR Biomedical Research Centre for Mental Health at the South London and Maudsley NHS Trust; funding for the two source datasets was provided by Pfizer Global Research and Development. The views expressed are those of the authors and not necessarily those of the NHS, the NIHR, or the Department of Health and Social Care.

## PATIENT CONSENT STATEMENT

Written informed consent was obtained from all subjects to participate in the study.

## Supporting information


**Appendix S1** Supporting Information

## Data Availability

Pain and control imaging data that support the findings of this study are available on request from the corresponding author. The data are not publicly available due to privacy or ethical restrictions.
