# Supplementary material for: Neurotransmitter receptor densities are associated with changes in regional Cerebral blood flow during clinical ongoing pain
Source: Hum Brain Mapp. 2022 Jul 7;43(17):5235–49. doi: 10.1002/hbm.25999 (PMC9812236; doi:10.1002/hbm.25999)
Supplement: Supplementary file 1 — Appendix S1 Supporting Information [file HBM-43-5235-s001.docx]

***Supplemental Materials***

**PART A.**

1. **Third Molar Extraction (TME) participants inclusion and exclusion criteria:**

## Inclusion Criteria

Subjects must meet all of the following inclusion criteria to be eligible for enrolment into the study:

- Right-handed male subjects with established painful intermittent pericoronitis, between the ages of 18 and 50 years, inclusive.
- Contralateral matched mandibular third molars with pericoronitis that require extraction (NICE guidelines 2000). Otherwise full healthy dentition.
- Body Mass Index (BMI) of approximately 18 to 30 kg/m2; and a total body weight 50-100 kg.
- Ongoing pain VAS score less than one on admission to all sessions (NB- admission to session- not admission to post-surgical scanning)
- Evidence of a personally signed and dated informed consent document indicating that the subject (or a legally acceptable representative) has been informed of all pertinent aspects of the trial.
- Willing and able to comply with scheduled visits, treatment plan, laboratory tests, and other trial procedures.

## Exclusion Criteria

Subjects presenting with any of the following will not be included in the study:

- Prescribed oral nitrates.
- Tramadol or codeine within 12h before each session.
- NSAIDs or paracetamol within 12h before each session.
- History of psychosis or psychological disease either (a) requiring ongoing psychoactive drugs, or (b) that the Investigator has reason to believe will either affect the patient’s neural pathways or hinder the performance of the patient with regard to perception of pain or ability to successfully complete the tasks required of them according to the protocol.
- Any person unable to lie still within the environment of the fMRI scanner for the required period to perform the study and those where MRI scanning is contraindicated (metal, pacemaker, etc).
- Any person unable to understand and follow the instructions of the investigators.
- Any evidence of a history or current use of drugs of abuse.
- Unwilling or unable to conform to lifestyle guidelines.
- Existing problems, for example, uncontrolled hypertension, renal failure, cancer, liver disease, severe spinal trauma, active thyroid disease, congestive heart failure, etc.
- Known history of other disorder which is strongly associated with polyneuropathy, including alcohol.
- Non-caucasian ethnicity.
- Subjects that smoke more than 5 cigarettes per day or consume more than 6 cups of caffeinated drinks per day.

## Life Style Guidelines

Subjects will abstain from alcohol for 24 hours prior to each session.

Subjects will abstain from caffeine-containing products for 6 hours prior to each session.

Subjects will abstain from the use of tobacco- or nicotine-containing products for 4 hours prior to admission until discharge for each session.

1. **Osteoarthritis patients (OA) and Controls inclusion and exclusion criteria:**

## Inclusion Criteria

Subjects must meet all of the following inclusion criteria to be eligible for enrolment into the study:

- Female, any race, right handed, at least 18 -80 years of age;
- [Patient Group Only] The clinical diagnosis of osteoarthritis of the dominant hand, according to American College of Rheumatology guidelines, of at least 6 months duration.
- [Patient Group Only] Pain around one of the first carpometacarpal (CMC) joints due to the osteoarthritis. The patient must report the average pain in this joint in the week prior to screening as at least 3 on an 11-point numerical rating scale at the time of screening.
- Evidence of a personally signed and dated informed consent document indicating that the subject has been informed of all pertinent aspects of the trial.
- Willing and able to comply with scheduled visits and trial procedures.

## Exclusion Criteria

Subjects presenting with any of the following will not be included in the study:

- Subjects with a body mass index (BMI) less than 18 or greater than 35 kg/m^2^;
- Subjects that smoke more than 5 cigarettes per day or consume more than 6 cups of caffeinated drinks per day;
- History of psychosis or psychological disease requiring ongoing psychoactive drugs (excluding anti-depressants stable in dose for at least 3 months and not anticipated to change);
- Any person unable to lie still within the environment of the fMRI scanner for the required period to perform the study and those where MRI scanning is contraindicated (metal, pacemaker, etc);
- Clinically significant or unstable medical or psychological conditions that, in the opinion of the investigator, would compromise participation in the study;
- Other severe pain which in the opinion of the investigator would impair the assessment of the pain due to osteoarthritis and thus may compromise participation in the study;
- Skin condition over the CMC joint which will be used for pain threshold testing, that could interfere with the assessment of pain thresholds;
- Acute joint trauma of the hand within 12 months of commencing the study
- Use of prohibited medications as listed below, in the absence of appropriate washout periods.
- Oral or intramuscular corticosteroids within 4 weeks prior to screening
- Monoamine oxidase inhibitors within 2 weeks of screening
- Analgesic agents, other than NSAIDs, Cox-2 inhibitors, or acetaminophen within 1 week prior to screening. Aspirin use ≤ 325 mg per day for cardiovascular prophylaxis is permitted. The use of NSAIDs ,Cox-2 inhibitors and compound analgesic medications (containing low-dose opioid components) is permitted provided the patient is on a stable regimen for at least 4 weeks prior to screening and for the duration of the study. Acetaminophen up to 4 g/day may be used, provided it is not used in the 24 hours prior to each session
- Intra-articular steroids into the study joint within 12 weeks, and to any other joint within 4 weeks prior to screening.

## Life Style Guidelines

Subjects will abstain from alcohol for 24 hours prior to each session.

Subjects will abstain from caffeine-containing products for 6 hours prior to each session.

Subjects will abstain from the use of tobacco- or nicotine-containing products for 4 hours prior to admission until discharge for each session.

**PART B.**


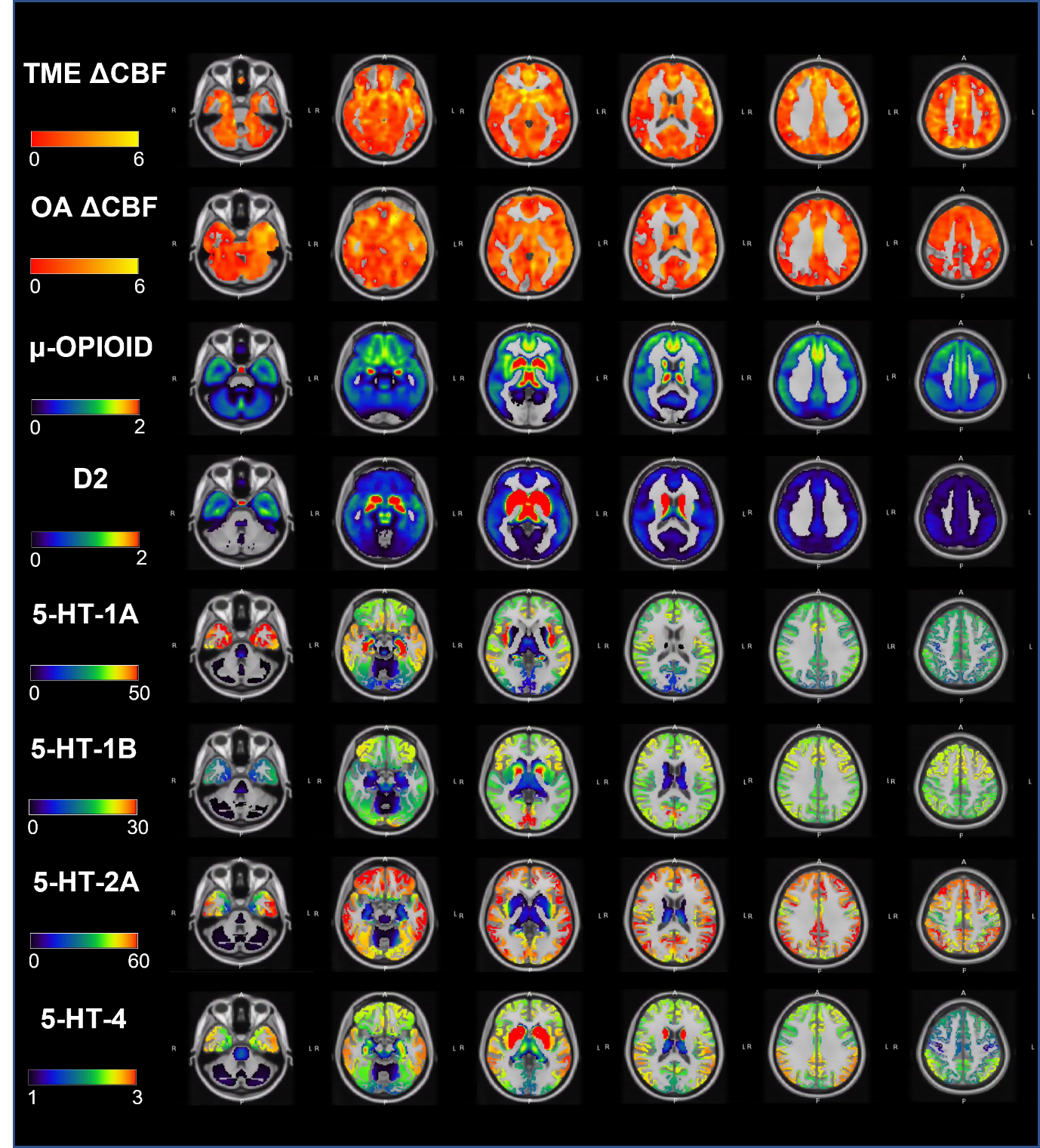


**Supplemental Figure 1.** A sample of axial slices presenting the pain vs non-pain for TME, and OA vs Controls ΔCBFs in terms of T-score maps (top two rows) and receptor BPnd PET maps (bottom six rows) that were used in the linear correlation analysis. All maps are presented after white matter masking out, while BPnd maps are presented prior to applying any normalization or log-transformation.


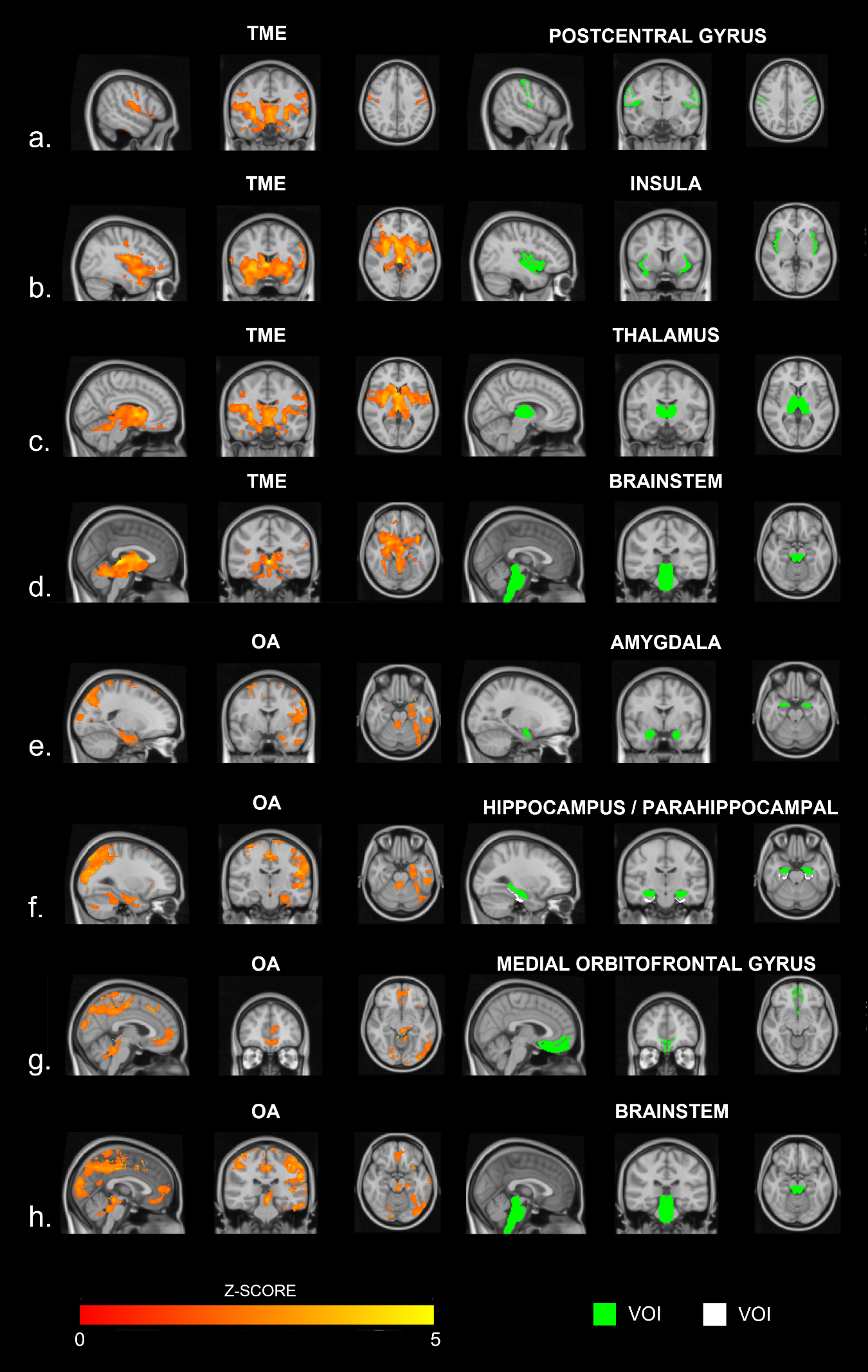


**Supplemental Figure 2.** A sample of brain slices in the three planes showing statistically significant increases in CBF of pain compared to non-pain TME, and OA compared to Controls groups, in terms of cluster-corrected Z-score maps (left), along with the corresponding Desikan-Killiany atlas VOIs (green/white) with which they present a significant overlap (right). ΔCBF significant increases of **TME** pain are shown for: (**a.**) Postcentral Gyrus (featuring overlap with primary and secondary Somatosensory cortices); (**b.**) Insula; (**c.**) Thalamus and (**d.**) Brainstem (featuring overlap with Midbrain/Periaqueductal Gray). ΔCBF significant increases of **OA** are shown for: (**e.**) Amygdala; (**f.**) Hippocampus (green) and Parahippocampal Gyrus (white); (**g.**) Medial Orbitofrontal Gyrus and (**h.**) Brainstem (featuring overlap with Midbrain/Periaqueductal Gray)

**PART C.**

**Supplemental Table 1**

| **No** | **VOI NAME** | **POSITION** | **TME ΔCBF**  Average T-score | **OA ΔCBF**  Average T-score |
| --- | --- | --- | --- | --- |
| 1 | lh_caudalanteriorcingulate | frontal lobe | 3.48 | 2.73 |
| 2 | lh_caudalmiddlefrontal | frontal lobe | 2.34 | 1.92 |
| 3 | lh_cuneus | occipital lobe | 2.09 | 1.27 |
| 4 | lh_entorhinal | temporal lobe | 2.30 | 2.27 |
| 5 | lh_fusiform | temporal lobe | 1.54 | 2.17 |
| 6 | lh_inferiorparietal | parietal lobe | 2.07 | 1.75 |
| 7 | lh_inferiortemporal | temporal lobe | 1.49 | 2.53 |
| 8 | lh_isthmuscingulate | frontal lobe | 2.69 | 1.82 |
| 9 | lh_lateraloccipital | occipital lobe | 1.09 | 1.67 |
| 10 | lh_lateralorbitofrontal | frontal lobe | 1.56 | 2.74 |
| 11 | lh_lingual | occipital lobe | 1.99 | 2.02 |
| 12 | lh_medialorbitofrontal | frontal lobe | 3.16 | 1.76 |
| 13 | lh_middletemporal | temporal lobe | 1.65 | 2.70 |
| 14 | lh_parahippocampal | temporal lobe | 1.57 | 2.81 |
| 15 | lh_paracentral | parietal lobe | 2.84 | 1.43 |
| 16 | lh_parsopercularis | frontal lobe | 2.62 | 2.36 |
| 17 | lh_parsorbitalis | frontal lobe | 2.14 | 1.89 |
| 18 | lh_parstriangularis | frontal lobe | 2.09 | 2.09 |
| 19 | lh_pericalcarine | occipital lobe | 1.91 | 1.72 |
| 20 | lh_postcentral | parietal lobe | 3.10 | 1.75 |
| 21 | lh_posteriorcingulate | parietal lobe | 2.84 | 2.31 |
| 22 | lh_precentral | frontal lobe | 2.75 | 2.13 |
| 23 | lh_precuneus | parietal lobe | 1.92 | 0.72 |
| 24 | lh_rostralanteriorcingulate | frontal lobe | 3.89 | 1.92 |
| 25 | lh_rostralmiddlefrontal | frontal lobe | 2.27 | 1.60 |
| 26 | lh_superiorfrontal | Frontal lobe | 2.74 | 1.87 |
| 27 | lh_superiorparietal | parietal lobe | 3.17 | 0.56 |
| 28 | lh_superiortemporal | temporal lobe | 2.06 | 2.47 |
| 29 | lh_supramarginal | parietal lobe | 2.77 | 1.70 |
| 30 | lh_frontalpole | frontal lobe | 1.45 | 2.21 |
| 31 | lh_temporalpole | temporal lobe | 2.15 | 2.22 |
| 32 | lh_transversetemporal | temporal lobe | 1.36 | 2.41 |
| 33 | lh_insula | frontal lobe | 2.69 | 2.09 |
| 34 | rh_caudalanteriorcingulate | frontal lobe | 2.93 | 2.92 |
| 35 | rh_caudalmiddlefrontal | frontal lobe | 1.86 | 1.51 |
| 36 | rh_cuneus | occipital lobe | 1.93 | 0.64 |
| 37 | rh_entorhinal | temporal lobe | 2.56 | 0.60 |
| 38 | rh_fusiform | temporal lobe | 1.96 | 0.97 |
| 39 | rh_inferiorparietal | parietal lobe | 1.74 | 0.54 |
| 40 | rh_inferiortemporal | temporal lobe | 1.36 | 1.02 |
| 41 | rh_isthmuscingulate | frontal lobe | 2.17 | 1.43 |
| 42 | rh_lateraloccipital | occipital lobe | 1.21 | 0.50 |
| 43 | rh_lateralorbitofrontal | frontal lobe | 2.04 | 2.79 |
| 44 | rh_lingual | occipital lobe | 1.89 | 1.12 |
| 45 | rh_medialorbitofrontal | frontal lobe | 2.75 | 1.66 |
| 46 | rh_middletemporal | temporal lobe | 1.48 | 1.44 |
| 47 | rh_parahippocampal | temporal lobe | 2.69 | 1.14 |
| 48 | rh_paracentral | parietal lobe | 2.49 | 0.59 |
| 49 | rh_parsopercularis | frontal lobe | 2.57 | 1.27 |
| 50 | rh_parsorbitalis | frontal lobe | 2.18 | 1.84 |
| 51 | rh_parstriangularis | frontal lobe | 2.40 | 1.77 |
| 52 | rh_pericalcarine | occipital lobe | 1.91 | 0.70 |
| 53 | rh_postcentral | parietal lobe | 2.43 | 0.44 |
| 54 | rh_posteriorcingulate | parietal lobe | 2.22 | 2.05 |
| 55 | rh_precentral | frontal lobe | 2.25 | 1.00 |
| 56 | rh_precuneus | parietal lobe | 1.89 | 0.39 |
| 57 | rh_rostralanteriorcingulate | frontal lobe | 3.41 | 1.55 |
| 58 | rh_rostralmiddlefrontal | frontal lobe | 2.48 | 1.61 |
| 59 | rh_superiorfrontal | frontal lobe | 2.78 | 1.45 |
| 60 | rh_superiorparietal | parietal lobe | 2.86 | 0.31 |
| 61 | rh_superiortemporal | temporal lobe | 2.02 | 1.51 |
| 62 | rh_supramarginal | parietal lobe | 1.47 | 0.19 |
| 63 | rh_frontalpole | frontal lobe | 1.76 | 2.46 |
| 64 | rh_temporalpole | temporal lobe | 2.96 | 1.69 |
| 65 | rh_transversetemporal | temporal lobe | 2.25 | 0.93 |
| 66 | rh_insula | frontal lobe | 3.09 | 1.45 |
| 67 | lh_cerebellum | cerebellum | 1.64 | 1.94 |
| 68 | lh_thalamus | thalamus | 2.57 | 2.84 |
| 69 | lh_caudate | caudate | 3.49 | 2.70 |
| 70 | lh_putamen | putamen | 3.56 | 2.40 |
| 71 | lh_pallidum | pallidum | 3.61 | 2.14 |
| 72 | brainstem | brainstem | 2.44 | 1.12 |
| 73 | lh_hippocampus | hippocampus | 1.84 | 2.71 |
| 74 | lh_amygdala | amygdala | 2.40 | 2.55 |
| 75 | lh_Accumbens | accumbens | 3.42 | 2.82 |
| 76 | lh_ventraldiencephalon | thalamus | 2.67 | 2.39 |
| 77 | rh_cerebellum | cerebellum | 2.20 | 1.09 |
| 78 | rh_thalamus | thalamus | 2.14 | 1.79 |
| 79 | rh_caudate | caudate | 3.93 | 2.47 |
| 80 | rh_putamen | putamen | 3.14 | 2.27 |
| 81 | rh_pallidum | pallidum | 2.42 | 1.84 |
| 82 | rh_hippocampus | hippocampus | 2.17 | 1.09 |
| 83 | rh_amygdala | amygdala | 2.80 | 0.86 |
| 84 | rh_Accumbens | accumbens | 3.47 | 1.93 |
| 85 | rh_ventraldiencephalon | thalamus | 2.57 | 1.64 |

*lh, Left Hemisphere; rh, Right Hemisphere.
